# Department of the Interior, Census Office

**Published:** 1889-06

**Authors:** Robert L Porter

**Affiliations:** Superintendent of Census.


DEPARTMENT OF THE INTERIOR, CENSUS OFFICE:
WASHINGTON, D. C., May 1, '89.

*To the Medical Profession:—*

The various medical associations and the medical profession will be glad to learn that Dr. John S. Billings, Surgeon U. S. Army, has consented to take charge of the Report on the Mortality and Vital Statistics of the United States as returned by the Eleventh Census.

As the United States has no system of registration of vital statistics, such as is relied upon by other civilized nations for the purpose of ascertaining the actual movement of population, our census affords the only opportunity of obtaining near an approximate estimate of the birth and death rates of much the larger part of the country, which is entirely unprovided with any satisfactory system of State and municipal registration.

In view of this, the Census Office, during the month of May this year, will issue to the medical profession throughout the country "Physician's Registers" for the purpose of obtaining more accurate returns of deaths than it is possible for the enumerators to make. It is earnestly hoped that physicians in every part of the country will co-operate with the Census Office in this important work. The record should be kept from June 1, 1889, to May 31, 1890. Nearly 26,000 of these registration books were filled up and returned to the office in 1880, and nearly all of them used for statistical purposes. It is hoped that double this number will be obtained for the Eleventh Census.

Physicians not receiving Registers can obtain them by sending their names and addressss to the Census Office, and, with the Register, an official envelope which requires no stamp will be provided for their return to Washington.

If all medical and surgical practitioners throughout the country will lend their aid, the mortality and vital statistics of the Eleventh Census will be more comprehensive and complete than they have ever been. Every physician should take a personal pride in having this report as full and accurate as it is possible to make it.

It is hereby promised that all information obtained through this source shall be held strictly confidential.

ROBERT L PORTER,
Superintendent of Census.

